# Neuronal MicroRNA Deregulation in Response to Alzheimer's Disease Amyloid-β

**DOI:** 10.1371/journal.pone.0011070

**Published:** 2010-06-11

**Authors:** Nicole Schonrock, Yazi D. Ke, David Humphreys, Matthias Staufenbiel, Lars M. Ittner, Thomas Preiss, Jürgen Götz

**Affiliations:** 1 Alzheimer's and Parkinson's Disease Laboratory, Brain and Mind Research Institute, University of Sydney, Sydney, New South Wales, Australia; 2 Molecular Genetics Division, Victor Chang Cardiac Research Institute (VCCRI), Darlinghurst, Sydney, New South Wales, Australia; 3 Novartis Institute for BioMedical Research, Basel, Switzerland; 4 School of Biotechnology and Biomolecular Sciences and St. Vincent's Clinical School, University of New South Wales, Sydney, New South Wales, Australia; Brigham and Women's Hospital, Harvard Medical School, United States of America

## Abstract

Normal brain development and function depends on microRNA (miRNA) networks to fine tune the balance between the transcriptome and proteome of the cell. These small non-coding RNA regulators are highly enriched in brain where they play key roles in neuronal development, plasticity and disease. In neurodegenerative disorders such as Alzheimer's disease (AD), brain miRNA profiles are altered; thus miRNA dysfunction could be both a cause and a consequence of disease. Our study dissects the complexity of human AD pathology, and addresses the hypothesis that amyloid-β (Aβ) itself, a known causative factor of AD, causes neuronal miRNA deregulation, which could contribute to the pathomechanisms of AD. We used sensitive TaqMan low density miRNA arrays (TLDA) on murine primary hippocampal cultures to show that about half of all miRNAs tested were down-regulated in response to Aβ peptides. Time-course assays of neuronal Aβ treatments show that Aβ is in fact a powerful regulator of miRNA levels as the response of certain mature miRNAs is extremely rapid. Bioinformatic analysis predicts that the deregulated miRNAs are likely to affect target genes present in prominent neuronal pathways known to be disrupted in AD. Remarkably, we also found that the miRNA deregulation in hippocampal cultures was paralleled in *vivo* by a deregulation in the hippocampus of Aβ42-depositing APP23 mice, at the onset of Aβ plaque formation. In addition, the miRNA deregulation in hippocampal cultures and APP23 hippocampus overlaps with those obtained in human AD studies. Taken together, our findings suggest that neuronal miRNA deregulation in response to an insult by Aβ may be an important factor contributing to the cascade of events leading to AD.

## Introduction

Alzheimer's disease (AD) is a prominent neurodegenerative disorder characterized by progressive loss of memory and other cognitive functions. Histopathologically, AD is characterized by neurofibrillary tangles (NFTs) consisting of the microtubule-associated protein tau and neuritic plaques composed of amyloid-β (Aβ). Aβ is a naturally occurring, predominantly 40 amino acid long polypeptide (Aβ40) derived from the larger amyloid precursor protein (APP) [1]. Increases in the proportion of the longer, more neurotoxic form, Aβ42, result in the formation of higher order aggregates and subsequently, plaque deposition. In familial AD (FAD), the increases in Aβ42 are caused by aberrant processing of APP due to mutations in either the *APP* gene itself or in genes that encode subunits of the APP processing machinery. In addition, *APP* promoter polymorphisms [2], gene duplications [3] or trisomy 21 [4] can cause increased *APP* expression levels, resulting in elevated Aβ42. While increased Aβ levels characterize AD pathology, the precise mechanism(s) and signaling cascades it uses to cause cellular toxicity and cell death are not fully understood [5], [6].

To better understand disease initiation and progression, transgenic animal models have been developed that model aspects of AD [7]. APP23 mice over-express the FAD mutant human APP in brain, and develop amyloid plaques similar to the human pathology [8]. These mice mimic several of the histopathological, biochemical, cognitive and behavioral alterations characteristic for AD. More recently, the research focus has shifted away from plaque formation to earlier events in disease progression such as the deregulation of genes whose impact on disease is still largely unknown [9]. A substantial portion of post-transcriptional gene regulation is controlled by microRNA (miRNA) networks, hence an alteration in the expression of miRNAs is emerging as a significant contributing factor to human neurodegenerative disease [10], [11]. miRNAs are evolutionarily conserved non-coding RNAs of ∼22 nucleotides that negatively regulate gene expression in a sequence-specific manner. Indeed, profiling of postmortem human AD brain has verified that significant changes in miRNA expression occur in several brain regions [10]. This includes miRNAs that regulate genes such as *APP* itself, and *BACE1*, that encodes an enzyme involved in APP processing [12], [13], [14]. However, whether the deregulated miRNAs are a cause or a consequence of disease, and what triggers miRNA dysfunction in AD is unknown. We therefore explored the hypothesis that Aβ itself causes neuronal miRNA deregulation which could contribute to the pathology associated with AD. To remove the complexity inherently associated with human studies, we used mature murine primary hippocampal cultures to determine the effects of Aβ specifically on neuronal miRNAs.

Sensitive TaqMan low density miRNA arrays (TLDA) revealed that 47% of all miRNAs tested were down-regulated in response to Aβ42. This response may be extremely rapid and bioinformatic analysis predicts that the deregulated miRNAs are likely to affect target genes present in prominent neuronal pathways disrupted in AD. Remarkably, when we analyzed hippocampi of APP23 mice at the onset of Aβ plaque formation, we found a similar miRNA deregulation as in our in *vitro* model. These findings support the notion that an insult by Aβ peptides causes a considerable neuronal miRNA deregulation that may be an important factor in the pathocascade of events leading to AD.

## Materials and Methods

### Ethics Statement

All animal experiments were approved by the Animal Ethics Committee (AEC) of the University of Sydney under AEC approval numbers K00/1-2009/3/4914 and K00/1-2009/3/4915.

### Cell culture and Aβ treatments

Primary hippocampal neurons were prepared from 16.5-day-old embryonic C57BL/6 mice (E16.5) as described [15]. 600,000 cells were plated per dish and cultivated in Neurobasal medium supplemented with 1% (v/v) B27 supplements (Gibco) and 0.25% (v/v) 200 mM L-glutamine (Gibco) to minimize growth of astrocytes and microglia. Synthetic Aβ42 peptides (Bachem, Germany) dissolved in PBS were aged by incubation at 37°C for 24 h with shaking at 1000 rpm to allow fibril formation [16]. We applied a protocol as described which used a range of biophysical methods to determine the fibrillar nature of our preparation [17], [18]. At 23 days in vitro (DIV) cells were treated for 0, 1, 6, 15 or 24 hours with either 5 µM aged Aβ42 or a mock treatment containing PBS. Following treatments, cells were washed once in PBS and lysed by resuspension in QIAzol reagent (Qiagen). Cell lysates were stored in QIAzol at −80°C until further use. Experiments were performed in triplicate.

### Transgenic mouse strain

APP23 mice expressing human APP751 cDNA containing the Swedish double mutation (K651M and N652L) were used for this study [8]. Brains from mice at different ages (ranging from two to thirteen months) were harvested following cervical dislocation and the hippocampus was isolated, snap frozen and stored at −80°C until use. Hippocampi were not pooled and analysis was performed on individual animals. Non-transgenic littermates were used as controls.

### RNA extraction and microarray analysis

RNA was extracted from mouse primary hippocampal neurons and dissected hippocampi using the miRNeasy Kit (Qiagen) according to the manufacturer's instructions. RNA quantity was routinely assessed on a NanoDrop 1000 spectrophotometer (Thermo Scientific). For microarray analysis, RNA quality was determined on a Bioanalyser 2100 (Agilent) and only RNA samples with an RNA integrity number (RIN) between 8 and 10 were used. Megaplex profiling using rodent TaqMan Low Density miRNA Arrays (TLDA) (Applied Biosystems) was used to assay the expression of 380 miRNAs as described by the manufacturer. Briefly, 100 ng of total RNA obtained from Aβ42- or control-treated primary hippocampal cells was used in the megaplex reverse transcription (RT) reaction containing about 450 miRNA-specific RT primers provided by the manufacturer. No prior miRNA preamplification step was needed. The RT product was mixed with 2X TaqMan Universal PCR Master Mix, No AmpErase UNG (Applied Biosystems) and loaded onto the TLDA containing the 48-plex PCR reaction mix. TLDAs were run on a 7900HT Thermocycler (Applied Biosystems) using Sequence Detection Systems (SDS) software version 2.3. A single TLDA was used per Aβ- or control-treated sample. Manual inspection of all amplification plots was performed and miRNAs were excluded from the analysis if: C*t* values were too high (above 35, indicating a miRNA expression too low for accurate detection), if amplification was not achieved in all six samples, or if very high variation was found. Data analysis was performed using SDS RQ manager v1.2 (Applied Biosystems) which utilizes the delta-delta CT method [19]. The endogenous small nucleolar control RNA, snoRNA234, was used for normalization. Significance was calculated using the student's T-test.

### Quantitative real-time PCR

Individual TaqMan assays (Applied Biosystems) were used to analyse the expression of the following mature mouse miRNAs: miR-181c, miR-9, miR-20b, miR-21, miR-30c, miR-148b, miR-361, miR-409-3p and Let-7i. 10 ng of total RNA was used in the RT reaction and the transcribed cDNA was then used for subsequent PCR amplification using TaqMan 2X Universal PCR Master Mix, No AmpErase UNG (Applied Biosystems) as described by the manufacturer. Assays were run on an Mx3000P thermocycler (Stratagene) as follows: 95°C for 10 min, and 40 cycles at 95°C for 151s followed by 60°C for 1 min. To avoid any miRNA degradation, RNA extractions, reverse transcription reactions and real-time runs were all performed on the same day. Mouse snoRNA135 expression was assayed for normalization. All reactions were performed in triplicate, and relative miRNA expression was normalized against endogenous controls using the comparative delta-delta CT method calculated using MxPRO Software V4.0 (Stratagene).

### In-*situ* hybridisation and immunohistochemistry

Ketamine/xylazine (Troy Laboratories, Australia) -anaesthetized mice were perfused with 20 ml PBS. Brain tissue was dissected and postfixed over night at 4°C in 4% paraformaldehyde (Sigma, Australia). Tissue embedding in paraffin was done in a Shandon Excelsior tissue processor (Thermo, USA). In-situ hybridization was performed as described [20]. Briefly, 15 µm paraffin-embedded sections of three month old mice were rehydrated, permeabilized with proteinase K (10 mg/ml for 5 min), and then refixed for 15 min in 4% PFA before hybridizing at 65°C overnight to a digoxigenin-labeled probe in a humidifying chamber. The 760 bp APP probe used for hybridization corresponds to the 3′ end of human APP751 following a BamHI digest. Slides were subsequently washed, prepared for immunohistochemistry with an alkaline phosphatase-conjugated antidigoxigenin antibody (Sigma), and developed in NBT/BCIP solution (Sigma).

For immunohistochemistry, antigen retrieval of 5 µm sections of APP23 and WT brain was performed in 10 mM citrate buffer, pH 5.8 in a RHS-1 microwave vacuum histoprocessor (Milestone, USA) at 120°C. For standardization, all stainings were carried out in Sequenza racks (Thermo, USA). Sections were blocked with PBS containing 3% heat inactivated goat serum and 5% BSA for 1 hour at room temperature followed by incubation at 4°C over night with the primary antibody 6E10 (1:1000, Signet, USA), which is reactive against amino acid residues 1-16 of Aβ. Antibody staining was visualized using the AP-ABC Elite Kit (Vector, USA).

### Immunocytochemistry

Coverslips containing mouse primary hippocampal neurons grown for 24 DIV were fixed with 4% paraformaldehyde in 80 mM PIPES, 1 mM MgCl2, and 1 mM EGTA, pH 6.8. Cells were permeabilized with 0.1% Triton in phosphate-buffered saline and stained with primary antibodies to rabbit β3 tubulin (1:400, Covance, USA). Antibody staining was visualized using Alexa labeled secondary antibodies (Molecular Probes, USA). Pictures were taken with a BX51 fluorescence microscope equipped with a DP70 CCD color camera (Olympus, USA).

### Pathway enrichment analysis of deregulated miRNAs

TargetScanMouse v5.1 [21] was used to generate a list of potential target genes for each of our significantly deregulated miRNAs found from the microarray analysis. Due to miRNA sequence similarities between family members, TargetScan mostly predicts target genes for miRNA families rather than for individual family members. We performed an enrichment analysis of target gene lists predicted for all significantly deregulated miRNAs using the DAVID (Database for Annotation, Visualization and Interrogated Discovery) bioinformatics database [22], [23]. Gene lists were uploaded into DAVID and enrichment analysis was performed by comparing each set of genes to all available biological pathways provided by the Kyoto Encyclopedia of Genes and Genomes (KEGG) [24]. A cut-off P-value of 0.01 was used to show KEGG Pathways likely to be affected by predicted targets of deregulated miRNAs.

## Results

### Neuronal miRNA expression changes upon exposure to amyloid-β

**Figure 1:**
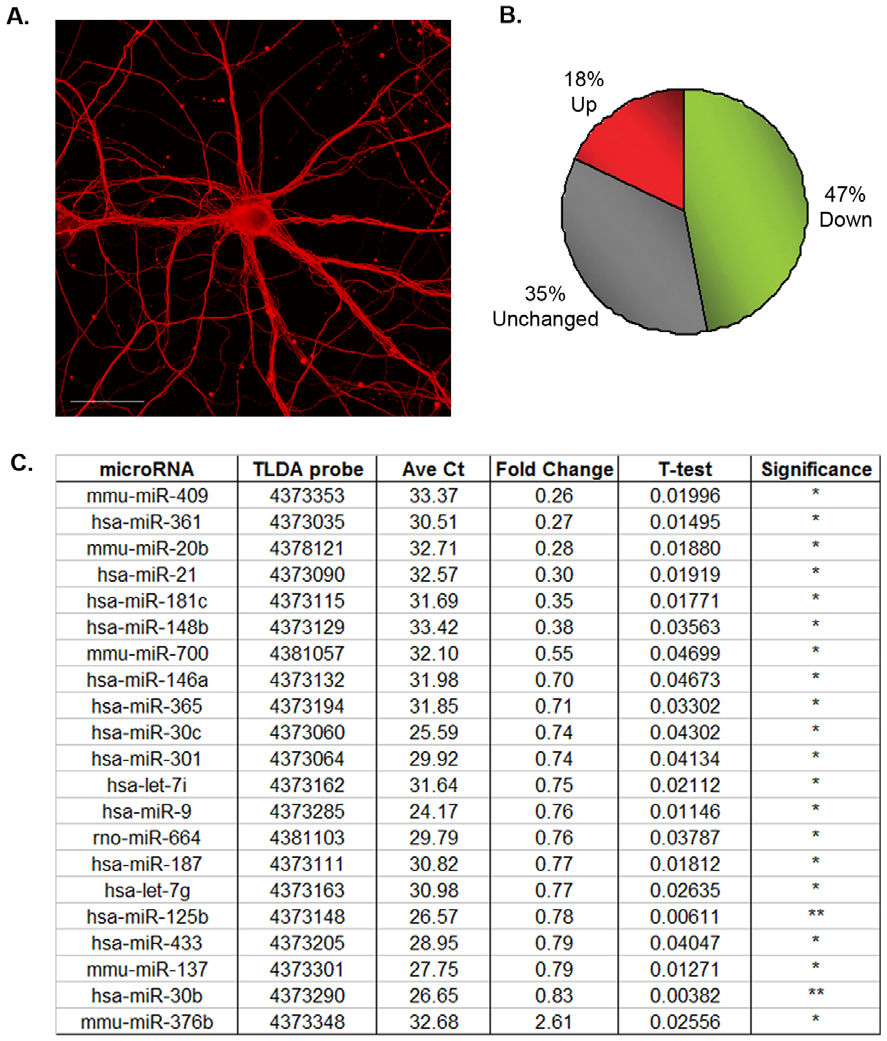
Deregulated miRNAs in mouse primary hippocampal cells treated with Aβ42. **A**. Mouse primary hippocampal neurons grown for 24 DIV (days in vitro) stained with neuronal β3 tubulin showing dense axonal networks indicative of healthy mature neurons. Scale bar  = 25 um. **B**. Neuronal miRNA response to Aβ treatment. Overview of directional miRNA changes after Aβ treatment. miRNAs altered by 15% compared to untreated controls were considered unchanged. **C**. Summary of significantly deregulated microRNAs in primary hippocampal cells with or without Aβ42 treatment (n = 3) analyzed by rodent TLDA. miRNA expression levels can be gauged using average (Ave) C*t* values. T-test P-value significance: **P<0.01, *P<0.05.

To determine whether neuronal miRNAs are deregulated in response to Aβ we used a cell culture model of murine primary hippocampal neurons [15]. Neurons were matured in *vitro* and neuronal β3 tubulin staining showed that they had developed dense axonal networks indicative of fully differentiated and healthy neurons (Fig 1A). At 23 DIV, triplicate cultures were exposed for 24 hours to either 5 µM of aged Aβ42 preparations or a PBS control. Under these conditions, no evidence of Aβ toxicity or cell death was observed. Propidium iodide (PI) uptake was negligible in Aβ-treated neurons indicative of viable cultures and β3 tubulin staining of Aβ-treated cells showed no neuronal fragmentation, an early sign of degeneration (data not shown). Total RNA was then isolated and expression of 381 miRNAs analyzed by qRT-PCR using rodent TaqMan Low Density miRNA Arrays (TLDA) (Applied Biosystems). Careful manual inspection of all amplification plots excluded miRNAs which did not amplify in all six samples, had very high variation, or had C*t* values above 35 indicating that their expression was too low for accurate analysis. Relative miRNA expression was normalized against the endogenous control snoRNA234 using the comparative delta-delta CT method as calculated using SDS RQ manager v1.2 software. For comparison, normalization was also performed using two other endogenous controls, snoRNA135 and 18S, included in each reverse transcription (RT) primer pool and the same end result was achieved (data not shown). 230 miRNAs (60%) were reliably detected on the array (Supplementary Table S1) of which 35% were considered unchanged as their expression levels varied only up to 15% from untreated controls. Interestingly, Aβ42 induced a considerable down-regulation of miRNAs with 47% showing decreased levels compared to untreated controls (Fig 1B). A much smaller fraction (18%) of miRNAs was up-regulated in response to Aβ42. When applying a cut-off P-value of <0.05, twenty miRNAs showed a significant down-regulation, of up to four fold, with the exception of miR-376b whose expression seemed to be strongly induced (2.6 fold) by Aβ treatment (Fig 1C). Amongst the strongest significantly down-regulated miRNAs were 409-3p, 361, 20b, 21, 181c and 148b. miRNAs 700, 146a, 365, 30c and 301 showed a moderate decrease, while miRNAs 9, 664, 187, 125b, 433, 137, 30b, Let-7i and Let-7g had a mild but significant down-regulation in response to Aβ. One advantage of using TaqMan assays is that their design, with three levels of specificity per miRNA, allows differentiation between mature miRNAs differing in only a few nucleotides. Despite the fact that the Let-7 family consists of eight members varying only by one to two nucleotides, only Let-7i, Let-7g and miR-98 were down-regulated by Aβ, whilst the others remained mostly unchanged. In addition, specific members of the miR-30 family (30c and 30b) were also significantly down-regulated in response to Aβ. Thus, treatment of mouse primary neurons with Aβ42 does indeed evoke a strong change in miRNA profiles with a substantial portion of miRNAs being down-regulated.

### Neuronal miRNA response to amyloid-β occurs rapidly

**Figure 2:**
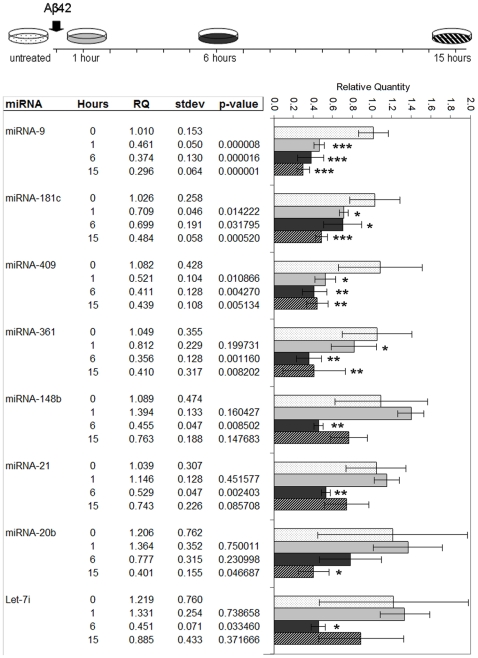
miRNA expression pattern in response to Aβ time course in murine primary hippocampal neurons assessed by real-time PCR using TaqMan assays. Independent primary neuronal preparations treated with Aβ42 for 1, 6 or 15 hours were assayed for miRNA expression relative to untreated controls. T-test P-value significance: ***P<0.001, **P<0.01, *P<0.05. Expression was normalized to snoRNA135.

Due to the low amounts of total RNA obtained from primary neuronal cells we chose to validate our microarray results by quantitative PCR using individual TaqMan assays on independent preparations of Aβ42-treated primary cultures. Eight significantly down-regulated miRNAs from the array data were selected for further validation and analysis. This selection was based on the fact that these miRNAs produced the most significant fold-changes in our study. In addition, an interesting overlap between human studies and ours was observed (miR-9, 181c, 30c, 148b, 20b and Let-7i) (Table 1) and therefore it was of great interest to validate and analyze these miRNAs in particular [13], [25]. miRNA expression was thus assayed in independent 24 DIV cultures treated with 5 µM aged Aβ42. As Aβ is known to cause cellular toxicity and cell death [26], a time course of 1, 6 and 15 hour treatments was used to exclude any effects due to apoptosis and to gauge the rapidity of the miRNA response evoked by Aβ. Similar to our TLDA microarray results, all of the eight tested miRNAs showed a significant down-regulation upon exposure to Aβ at some stage during the Aβ time-course treatment compared to untreated controls (Fig 2). Interestingly, Aβ caused an extremely rapid neuronal response of distinct mature miRNA sequences with miR-9, 181c, 409-3p and 361 responding even after a one hour Aβ treatment. Expression of miRNAs 148b, 21, 20b and Let-7i was more variable and therefore no conclusions can be made concerning the rapidity of their response to Aβ. Thus our analysis not only validates the microarray data but further shows that Aβ42 is a powerful regulator of certain mature miRNA sequences.

**Table 1:** Overlap in miRNA changes between Aβ42-treated murine hippocampal neurons and APP23 hippocampus found in this study and sporadic human AD brain.

	Mouse Neurons		APP23 Hippocampus		Human AD Brain			
miRNA	FC	P-value	FC	P-value	FC	P-value	Brain Region	Reference
miR-9	0.76	0.01146	0.54	0.00000002	0.71	0.0053	Anterior temporal cortex	[14]
					0.38 (–1.39*)	0.0231	Hippocampus, Braak 5,6	[26]
miR-181c	0.35	0.01771	0.66	0.00006444	0.71	0.0018	Anterior temporal cortex	[14]
					0.1 (–3.30*)	0.0015	Parietal lobe cortex	[51]
let-7i	0.75	0.02112	0.74	0.00000481	0.88	0.0283	Anterior temporal cortex	[14]
miR-30c	0.74	0.04302	0.72	0.00008285	0.3 (–1.76*)	0.0194	Hippocampus, Braak 3,4	[26]
miR-148b	0.38	0.03563	0.76	0.00139502	0.21 (–2.24*)	0.0062	Parietal lobe cortex	[51]
miR-20b	0.28	0.0188	0.80	0.25607460	0.28 (–1.84*)	0.0085	Parietal lobe cortex	[51]

Deregulated miRNA fold changes (FC) and significance (P-value) found in our study on Aβ-treated mouse primary neurons and APP23 hippocampus compared to those found in human AD profiling experiments on various brain regions (using the Braak staging). * FC values represented in the original reference from some human studies were in log form.

### Deregulated miRNAs may affect targets in key pathways altered in AD

A single miRNA is predicted to regulate several target genes, whilst a single gene can also be regulated by several miRNAs; hence changes in miRNA expression can have profound effects on biological systems [21]. To increase the likelihood of identifying biological processes most relevant to the miRNAs deregulated by Aβ we performed a gene ontology (GO) enrichment analysis on predicted target genes. Of the available prediction algorithms such as miRBase, PicTar, miRanda, PITA and TargetScan, all of which use site conservation as a prediction criterion, the latter was shown to result in the most accurate predictions upon target validation [27], [28]. Therefore, we used TargetScanMouse v5.1 to generate lists of predicted target genes regulated by our miRNAs of interest. Lists of target genes can be found at http://www.targetscan.org/mmu_50/. To extract biological meaning associated with these large gene lists we used the bioinformatics database DAVID. Pathway enrichment analysis was performed by comparing each list of target genes to all available biological pathways provided by the Kyoto Encyclopedia of Genes and Genomes (KEGG) [24]. Encouragingly, many pathways associated with brain function were enriched in the pathway prediction analysis (Table 2). *Axon guidance* was among the most significant pathways to be affected by the predicted target genes and was the top prediction for miR-9, miR-30 and miR-20. These three miRNAs that are down-regulated by Aβ potentially target a total of 36 out of 80 genes present in the *axon guidance* pathway, eight genes (*DPYSL2, DPYSL5, EPHA7, NFAT5, NFATC3, NTNG1, PPP3R1* and *SEMA6D*) may be targeted by two out of the three miRNAs, and *srGAP3* is predicted to be co-regulated by all three miRNAs. The other major pathway highlighted by this enrichment analysis is the *mitogen-activated protein kinase (MAPK) signaling pathway*. The MAPK cascade represents a prototypic signal transduction system through which extracellular stimuli are transduced. Three of the down-regulated miRNAs (miR-181, miR-21 and Let-7) have this pathway as their top candidate with p-values of less than 0.001. These three miRNAs alone are predicted to affect a total of 48 genes in the MAPK signaling pathway (containing 179 genes in total) with eight genes (*BRAF, FASl, MAP3K1, MAP4K4, TAOK1, TGFBR1, RASGRP1, RASA2* and *NLK*) co-targeted by two miRNAs and *ACVR1C*, encoding a serine/threonine protein kinase, predicted to be regulated by all three. In addition, categories such as *ErbB signaling* and *TGFβ signaling pathways* are targeted by several miRNAs as are pathways involved in apoptosis such as *ubiquitin mediated proteolysis*. Pathways essential for correct neuronal function such as *glutamate metabolism*, *long term potentiation* and *regulation of the actin cytoskeleton* are all enriched in our analysis and they are known to be disrupted in AD.

**Table 2:** Pathway enrichment analysis for deregulated miRNAs in mouse primary hippocampal neurons after Aβ treatment.

miRNA	Total Targets in mouse*	Targets assigned to KEGG pathways**	KEGG Pathway***	# Targets in Pathway****	P-value
miR-9	742	87	**Axon guidance**	**16 (80)**	**0.000150**
			Focal adhesion	17 (133)	0.004500
			Renal cell carcinoma	9 (48)	0.005800
			MAPK signaling pathway	20 (179)	0.006100
			Glutamate metabolism	6 (47)	0.006600
			ErbB signaling pathway	10 (60)	0.007700
			Regulation of actin cytoskeleton	17 (139)	0.009300
miR-30	871	69	**Axon guidance**	**16 (80)**	**0.000097**
			Ubiquitin mediated proteolysis	13 (118)	0.004800
miR-20b	741	56	**Axon guidance**	**14 (80)**	**0.000006**
			MAPK signaling pathway	14 (179)	0.006100
miR-181	639	83	**MAPK signaling pathway**	**23 (179)**	**0.000020**
			**Long-term potentiation**	**11 (42)**	**0.000032**
			**Dorso-ventral axis formation**	**7 (24)**	**0.000160**
			**T cell receptor signaling pathway**	**11 (80)**	**0.000950**
			Renal cell carcinoma	9 (48)	0.001600
			TGF-beta signaling pathway	10 (46)	0.002900
			Chronic myeloid leukemia	9 (51)	0.003300
			Colorectal cancer	9 (64)	0.006100
			mTOR signaling pathway	7 (30)	0.006800
			ErbB signaling pathway	9 (60)	0.007100
			Prostate cancer	9 (68)	0.007600
			Focal adhesion	14 (133)	0.010000
miR-21	143	23	**MAPK signaling pathway**	**10 (179)**	**0.000840**
			Colorectal cancer	5 (64)	0.010000
Let-7	683	72	**MAPK signaling pathway**	**26 (179)**	**0.000016**
			**Pancreatic cancer**	**11 (51)**	**0.000550**
			Bladder cancer	7 (33)	0.005200
			Glycan structures - biosynthesis	12 (115)	0.005700
			Melanoma	9 (38)	0.006300
			Chronic myeloid leukemia	9 (51)	0.010000
			Axon guidance	12 (80)	0.011000
miR-148b	368	53	Focal adhesion	13 (133)	0.001200
			Regulation of actin cytoskeleton	13 (139)	0.002300
			TGF-beta signaling pathway	8 (46)	0.004300
			Pancreatic cancer	7 (51)	0.006000
			Chronic myeloid leukemia	7 (51)	0.007200
miR-361	91	8	Huntington's disease	3 (143)	0.009000
miR-137	678	18	Glycosphingolipid biosynthesis	3 (25)	0.009400
miR-365	134	15	**Small cell lung cancer**	**6 (65)**	**0.001000**
			**Prostate cancer**	**6 (68)**	**0.001000**
			mTOR signaling pathway	5 (30)	0.001100
			Glioma	5 (39)	0.002200
			Melanoma	5 (38)	0.003400
			Focal adhesion	7 (133)	0.006900
miR-409-3p	101	1	P-value cutoff not met	∼	∼
miR-433	146	10	P-value cutoff not met	∼	∼
miR-376b	94	8	P-value cutoff not met	∼	∼
miR-146	85	0	No Pathways predicted	∼	∼
miR-700	No targets predicted	∼	∼	∼	∼
miR-187	No targets predicted	∼	∼	∼	∼
miR-664	No targets predicted	∼	∼	∼	∼
miR-125b	No targets predicted	∼	∼	∼	∼
miR-301	No targets predicted	∼	∼	∼	∼

*The total number of target genes predicted to be regulated by individual miRNAs was calculated using TargetScanMouse v5.1. Note that TargetScan gives lists of predicted targets for miRNA families and not for individual family members due to sequence similarity. **Gene lists were uploaded into DAVID bioinformatic database and the number of targets recognized by DAVID assigned to known KEGG pathways is given. ***Enrichment analysis showing KEGG Pathways likely to be affected by predicted targets of deregulated miRNAs are indicated using a cut-off P-value of 0.01. The most significant pathways with a P-value of 0.001 are shown in bold. ****For each pathway predicted to be affected the number of miRNA target genes in that pathway is indicated followed by the total number of genes in that pathway in brackets.

### miRNA down-regulation is paralleled in the APP23 mouse model

**Figure 3:**
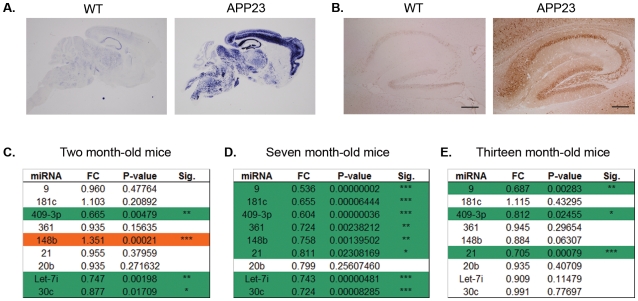
In *vivo* analysis of the Aβ plaque-forming APP23 mouse model. **A**. In *situ* hybridization showing *APP* expression pattern in brains of three month-old APP23 versus wild-type mice. Note the high expression of the transgene in cortex and hippocampus. **B**. Immunohistochemistry with the 6E10 antibody showing high levels of Aβ in the hippocampus of three month-old APP23 mice compared to littermate controls. Scale bar  = 500 µm. **C–E**. miRNA expression in hippocampus of two- (**C**.), seven- (**D**.) and thirteen- (**E**.) month-old APP23 mice compared to wild-type littermate controls (n = 4) represented as fold change (FC) as determined using real-time PCR with TaqMan assays. Down-regulated miRNAs have been highlighted in green and up-regulated ones in red. Reactions were normalized to snoRNA135 and P-value significance was calculated using the Students T-test: P-value  = *<0.05, **<0.01, ***<0.001.

To determine whether miRNA deregulation in response to Aβ also occurs in *vivo* we chose to analyze the well characterized APP23 mouse model [8]. The hippocampus was selected for analysis not only because the human APP mutant transgene is highly expressed in this brain region (Fig 3A) but it is also highly vulnerable to Aβ and it is where neuropathological changes are initiated early in AD [29]. The hippocampus also has a prominent role in learning and memory and its malfunction results in early memory loss and gradual decline of other cognitive functions characteristic of AD. Immunohistochemistry using the antibody 6E10 shows that Aβ is present at high levels in hippocampi of APP23 mice (Fig 3B). This was confirmed by ELISA analysis showing that both Aβ40 and Aβ42 are greatly overproduced in APP23 brain compared to WT (data not shown). In contrast to the published studies in humans which mostly show miRNA profiles at the end-stage of disease, we chose to analyze APP23 mice at various ages ranging from two month-old animals (pre-symptomatic, plaque-free stage), seven month-old (cognitive deficits apparent and onset of plaque deposition) and thirteen month-old mice (established AD pathology) [30], [31]. Utilizing their highly quantitative nature, individual TaqMan assays were used to analyze miRNA expression in hippocampi of four APP23 mice *versus* four wild-type littermate controls. In juvenile two month-old APP23 mice most of the miRNAs tested showed no change in expression levels compared to littermate controls. However, miR-409-3p, Let-7i and miR-30c already exhibited a significant down-regulation while miR-148b was the only up-regulated miRNA in hippocampus at this age (Fig 3C). As mice approach 6-7 months of age they reach the critical period of Aβ-plaque formation and it was here that a significant down-regulation was seen in nearly all miRNAs tested including the previously up-regulated miR-148b (Fig 3D). As mice continue to age only miRNAs 9, 409-3p and 21 maintain the significant down-regulation exhibited in seven month-old animals whilst the remaining miRNAs tested resume expression patterns similar to WT mice (Fig 3E). Thus the miRNA down-regulation found in Aβ42-treated hippocampal neurons was paralleled in *vivo* in hippocampi of APP23 mice, at the onset of plaque formation. Altogether, our studies reveal that Aβ is not only a powerful regulator of miRNAs in *vitro*, but also in *vivo*.

## Discussion

An important role for miRNAs has been shown in development and specifically in brain, where more distinct miRNAs are expressed than in any other tissue [32]; with evidence increasing for their implication in neurodegenerative disease [10], [11]. miRNA profiles are known to be altered in several regions of the AD brain, however what is cause or consequence of the disease is unknown [12], [13], [25], [33], [34], [35]. Our study dissects the complexity of early human AD pathogenesis, in the absence of a post mortem delay, and provides the first miRNA profiling analysis addressing the contribution of Aβ, a known causative factor of AD, to the de-regulation of neuronal miRNA expression. We used both in *vitro* and in *vivo* models to show that Aβ evokes a substantial change in neuronal miRNA profiles that is not only predominantly down-regulated, but can occur rapidly, within a few hours of Aβ treatment. Several of these deregulated miRNAs overlap with those found in human AD studies and potentially affect important biological pathways essential for proper brain function relevant to AD.

miRNAs seem to provide essential neuroprotective functions, as decreases result in neurodegeneration [36], [37], [38]. Importantly, this paradigm is reiterated in our study where Aβ predominantly causes a down-regulation of miRNAs in hippocampal neurons. We also found that this down-regulation of mature miRNAs is extremely rapid for several miRNAs tested and is paralleled in *vivo* in the hippocampus of the APP23 AD mouse model at the onset of plaque formation.

Although changes in miRNA levels have been linked to several disease states such as Parkinson's disease [36], Huntington's disease [39], [40], schizophrenia [41] and Down's syndrome [42], the mechanisms responsible for stabilized or reduced miRNA expression have remained largely elusive. The biological effects of miRNAs are coordinated by the abundance of mature miRNA molecules and accumulation of a specific miRNA depends on the rates of transcription, processing and decay. There are several possible mechanisms that would explain the rapid down-regulation of certain mature miRNAs observed in Aβ-treated neurons:

1) Substantial nucleotide substitutions, additions and deletions have been detected in animal miRNAs [43], [44]. Although the sensitivity and specificity of TaqMan assays is far greater than for spotted arrays, each assay is highly specific for a predefined, mature miRNA sequence. Thus, any changes in sequence or length such as uridylation or adenylation of 3′ ends will interfere with TaqMan detection and may result in a down-regulated read-out [45], [46]. 2) In brain, miRNA turn-over may be rapid, and Aβ may interfere with the multiple steps involved in the production of mature, ∼22 nt miRNAs [47]. That the stability of mature miRNAs varies considerably was shown for the highly abundant, hepatocyte-specific microRNA miR-122 (T_1/2_>24 hrs) [48], while several brain-enriched miRNAs, such as miR-9, 125b, 146a, 132 and 183 exhibit short half-lives ranging from 1 to 3.5 hrs [35]. The decay rates for miR-9 are comparable in human brain tissue (T_1/2_ = 48 min) and neuronal cells in culture (T_1/2_ = 42 min), highlighting the validity of the in *vitro* model used by us. 3) Aβ may induce a rapid activation of miRNA-specific nucleases such as XRN-2, a 5′ to 3′ exonuclease [49]. However, whether XRN-2 or other unknown nucleases are up-regulated in AD remains to be determined. 4) Incorporation into Argonaute-containing protein complexes protects mature miRNAs from exonucleolytic pathways [50]. Target availability also affects miRNA release from Argonaute, which may be triggered by Aβ, resulting in subsequent degradation [49].

5) Finally, miRNAs are not only required during development but are essential to maintain function of the adult brain, e.g. at the synapse [51]. Aβ-induced miRNA regulation may involve alterations in cellular localization that will not only impact function but also turnover rate of miRNAs [52].

Interestingly, in our study, most of the mature miRNAs shown to be down-regulated in the Aβ time-course assay were also down-regulated in *vivo* in hippocampi of APP23 mice. Aβ levels are not as high in mouse brain as those used to acutely treat hippocampal cultures, but never the less a strong decrease in miRNA expression was also seen in seven month-old APP23 mice. At this age, mice reach the critical period of Aβ plaque formation where insoluble Aβ42 peptides increase five-fold compared to younger animals and the first scarce small plaques can be seen in hippocampus and neocortex. In addition, the mice display major cognitive deficits affecting visuo-spatial learning abilities [31]. Not many miRNAs were altered in two month-old APP23 mice, representing a juvenile age prior to deposition of Aβ plaques and in which no changes in behavioral performance is evident. Similarly, most miRNAs were comparable between APP23 and WT at thirteen months of age where AD pathology is evident with plaque deposits having increased in size and number and most histopathological, biochemical, cognitive and behavioral alterations characteristic for AD being present. Interestingly, miR-9 maintained its down-regulation in older mice and miR-409-3p was the only miRNA to be consistently down-regulated in APP23 from a very young age right through to older animals. Normalization of miRNA levels in thirteen month-old mice may be the result of several mechanisms including the induction of compensatory physiological responses induced by prolonged acute over-expression of proteins in transgenic mice or qualitative changes in Aβ such as aggregation of Aβ into oligomeric or fibrillar species. However, the exact mechanisms responsible for these patterns of miRNA deregulation occurring in *vivo* are likely complex and remain to be determined.

Of great interest is the possible overlap in miRNA deregulation between the models used in our study with the existing profiling studies performed on human AD brain (Table 1). In general, miRNA expression studies on AD patients revealed either no or only very little overlap in miRNA changes (reviewed in [10]). However, similar to our finding, Hebert et al showed that in AD temporal cortex the deregulated miRNAs were also mostly down-regulated compared to controls [13]. Importantly, this human study showed that miR-9, 181c and Let-7i were down-regulated in AD brain. miR-9 has also been reported to be down-regulated in an independent human profiling study of various brain regions including hippocampus [25]. In addition, this study showed that miR-30c was down-regulated in hippocampus at an early stage of disease (Braak stages 3 and 4). A recent study by Nunez-Iglesias et al found forty-eight significantly deregulated miRNAs in human AD parietal lobe cortex, of which miR-148b, 20b and 181c were down-regulated [33]. Our in *vivo* analysis of APP23 hippocampus showed down-regulation of miR-9, 181c, 30c, 20b, 148b and Let-7i, all of which were altered in human AD brain. The overlap between human AD and our in *vitro* and in *vivo* AD models indicates that amongst the complex pathology in human AD brain, down-regulation of miR-9, miR-181c, miR-30c, miR-20b, miR-148b and Let-7i could be attributed at least in part to the presence of Aβ.

miR-9, the most abundant human brain miRNA [53], is a recurring candidate from several AD profiling studies. In contrast to the above studies including ours, miR-9 was found to be up-regulated in human AD CA1 [34] and temporal cortex [35]. Studies performed in zebrafish and mice revealed that miR-9 is essential in patterning, neurogenesis and differentiation and thus ideally placed to impact various aspects of brain function. Over-expression of miR-9 accelerates neuronal differentiation, while its inhibition in the medial pallium of E11.5 mouse embryos results in defective differentiation of Cajal-Retzius cells, the first neurons to populate the embryonic cortex. Similarly, loss of miR-9 in zebrafish embryos decreases the relative numbers of differentiated neurons in the anterior hindbrain [54], [55], [56]. Neurogenesis is not only important in the developing brain but is a process which continues in the adult hippocampus, a region heavily affected by Aβ pathology in AD [57]. Interestingly, AD patients exhibit altered expression of early neuronal markers in the hippocampus which has been attributed to increased neurogenesis [58]. Decreased expression of miR-9 may therefore impact adult brain function.

It is encouraging to see that most of the pathways predicted to be affected by miR-9 target genes are related to brain function. In comparison, miR-21, miR-181 and Let-7 have well characterized roles in cancer and it is not surprising therefore that their target genes result in enrichment for cancer-related pathways as well. The *MAPK pathway* was one of the top candidate pathways to be affected by Aβ-mediated down-regulation of miRNAs. This signaling cascade is involved in various cellular functions including hippocampal synaptic plasticity and learning. Indeed, even very low concentrations of oligomeric Aβ42 activate MAPK in human neuroblastoma cells [59]. In addition, MAPK activation was observed in hippocampal slice cultures of Aβ-forming Tg2576 mice [60]. In rodent hippocampus, MAPK is essential for LTP formation, and several APP mutant mouse strains exhibit deficits in hippocampal LTP and hippocampus-dependent associative learning paradigms, including contextual fear conditioning and escape training in the Morris water maze [30], [61], [62]. Thus, Aβ42-induced miRNA deregulation of the MAPK cascade may in part underlie the learning and memory deficits attributed to hippocampal dysfunction in AD.

*Axon guidance* was the other major pathway over-represented in our enrichment analysis. It represents a key stage in the formation of neuronal networks known to be disrupted in AD. The down-regulated miRNAs miR-9, miR-30 and miR-20 were all strongly predicted to affect target genes involved in axonal guidance. Interestingly, dihydropyrimidinase-related protein 2, *DPYSL2*, a highly abundant protein in brain, is targeted by miR-30, 20 and 181 and has been shown to be up-regulated in proteomic studies on APP23 mice already at a very early age [63]. Also called collapsin response mediator protein 2 (CRMP2), DPYSL2 is a signal mediator of Semaphorin 3A in the guidance of axonal growth. Dysregulation of DPYSL2 has also been reported in other AD proteomic studies [64], [65], [66], [67], along with its aberrant phosphorylation [68] and association with NFTs [69]. However, whether or not miRNAs play a role in its regulation remains to be determined.

Together, our work provides insight into previously unknown effects of Aβ on neuronal miRNA networks. We show that Aβ is a powerful regulator of miRNA expression, causing a rapid decrease of certain mature miRNA populations, which could have profound impacts on biological processes affecting the pathogenesis of AD. Aβ is well positioned to target several mechanisms that affect the stability of mature miRNAs, including alterations in cis-acting modifications, protein complex formation or the exposure to nucleases, to name a few [70]. However, the exact mechanism of the rapid Aβ-mediated down-regulation of mature brain miRNAs remains to be determined. The close overlap of our miRNA profiles in the cell culture model, APP23 hippocampus and human AD suggests the established APP23 mouse model is an ideal system to investigate further the role of Aβ-induced miRNA deregulation in AD. Our study uncovers an unexplored mechanism of how Aβ may impact the pathology of AD and the identification of key miRNAs affected by Aβ will allow further analysis of target genes and biological pathways contributing to pathomechanisms in AD.

## Supporting Information

Table S1 miRNA changes in mouse primary hippocampal cells evoked by Aβ42 treatment. Expression profiling of microRNAs in mouse primary hippocampal cells with and without Aβ42 treatment using Rodent TaqMan Low Denisty miRNA Arrays. Shown are the results for 230 miRNAs in ascending order out of the 381 miRNAs present on the TLDA whose amplification plots where above the cutoff threshold in the triplicate analysis. miRNA expression levels can be gauged using Average (Ave) C*t* values. miRNAs highlighted in bold are those significantly deregulated. T-test P-value significance: **P<0.01, *P<0.05.
(0.47 MB DOC)
