# Supplementary material for: Neuronal MicroRNA Deregulation in Response to Alzheimer's Disease Amyloid-β
Source: PLoS One. 2010 Jun 11;5(6):e11070. doi: 10.1371/journal.pone.0011070 (PMC2884018; doi:10.1371/journal.pone.0011070)
Supplement: Table S1 — miRNA changes in mouse primary hippocampal cells evoked by Aβ42 treatment. Expression profiling of microRNAs in mouse primary hippocampal cells with and without Aβ42 treatment using Rodent TaqMan Low Denisty miRNA Arrays. Shown are the results for 230 miRNAs in ascending order out of the 381 miRNAs present on the TLDA whose amplification plots where above the cutoff threshold in the triplicate analysis. miRNA expression levels can be gauged using Average (Ave) Ct values. miRNAs highlighted in bold are those significantly deregulated. T-test P-value significance: **P<0.01, *P<0.05. (0.47 MB DOC) [file pone.0011070.s001.doc]

**Supplemental Table S1.** miRNA changes in mouse primary hippocampal cells evoked by Aβ42 treatment.

| **microRNA** | **TLDA probe** | **Ave Ct** | **Fold Change** | **T-test** | **Significance** |
| --- | --- | --- | --- | --- | --- |
| snoRNA234 | 4380915 | 27.90 | normalizer |  |  |
| dme-let-7 | 4373493 | 30.88 | 0.94 | 0.6047 |  |
| hsa-let-7a | 4373169 | 30.61 | 0.93 | 0.7742 |  |
| hsa-let-7b | 4373168 | 30.44 | 0.92 | 0.5293 |  |
| hsa-let-7c | 4373167 | 28.39 | 0.99 | 0.8517 |  |
| hsa-let-7d | 4373166 | 32.23 | 1.10 | 0.7289 |  |
| hsa-let-7f | 4373164 | 30.06 | 1.21 | 0.7744 |  |
| **hsa-let-7g** | **4373163** | **30.98** | **0.77** | **0.0264** | ***** |
| **hsa-let-7i** | **4373162** | **31.64** | **0.75** | **0.0211** | ***** |
| hsa-miR-100 | 4373160 | 27.70 | 0.85 | 0.1004 |  |
| hsa-miR-101 | 4373159 | 32.91 | 1.15 | 0.4564 |  |
| hsa-miR-103 | 4373158 | 29.73 | 0.83 | 0.2031 |  |
| hsa-miR-106b | 4373155 | 33.19 | 0.76 | 0.2990 |  |
| hsa-miR-124a | 4373150 | 28.84 | 0.92 | 0.3849 |  |
| hsa-miR-125a | 4373149 | 27.27 | 0.93 | 0.5515 |  |
| **hsa-miR-125b** | **4373148** | **26.57** | **0.78** | **0.0061** | ****** |
| hsa-miR-126 | 4378064 | 29.54 | 0.79 | 0.2449 |  |
| hsa-miR-126# | 4373269 | 34.48 | 2.66 | 0.0648 |  |
| hsa-miR-127 | 4373147 | 27.88 | 0.88 | 0.2229 |  |
| hsa-miR-130a | 4373145 | 31.87 | 0.89 | 0.5625 |  |
| hsa-miR-130b | 4373144 | 32.64 | 5.13 | 0.5942 |  |
| hsa-miR-132 | 4373143 | 25.46 | 0.94 | 0.5208 |  |
| hsa-miR-133b | 4373172 | 35.27 | 1.15 | 0.8330 |  |
| hsa-miR-134 | 4373141 | 33.17 | 1.24 | 0.2234 |  |
| hsa-miR-135a | 4373140 | 32.04 | 0.85 | 0.3944 |  |
| hsa-miR-135b | 4373139 | 29.99 | 1.09 | 0.5333 |  |
| hsa-miR-136 | 4373173 | 35.37 | 2.01 | 0.3478 |  |
| hsa-miR-137 | 4373174 | 26.15 | 0.85 | 0.3832 |  |
| hsa-miR-139 | 4373176 | 29.39 | 1.03 | 0.8438 |  |
| hsa-miR-140 | 4373138 | 29.84 | 0.91 | 0.5104 |  |
| **hsa-miR-146a** | **4373132** | **31.98** | **0.70** | **0.0467** | ***** |
| hsa-miR-146b | 4373178 | 29.72 | 0.94 | 0.5452 |  |
| **hsa-miR-148b** | **4373129** | **33.42** | **0.38** | **0.0356** | ***** |
| hsa-miR-149 | 4373128 | 28.88 | 0.94 | 0.5627 |  |
| hsa-miR-152 | 4373126 | 31.69 | 0.29 | 0.0607 |  |
| hsa-miR-153 | 4373125 | 35.18 | 0.78 | 0.3386 |  |
| hsa-miR-15a | 4373123 | 32.21 | 1.39 | 0.7787 |  |
| hsa-miR-15b | 4373122 | 33.63 | 2.18 | 0.5506 |  |
| hsa-miR-16 | 4373121 | 26.24 | 0.93 | 0.5563 |  |
| hsa-miR-17-5p | 4373119 | 29.76 | 1.06 | 0.7725 |  |
| hsa-miR-181a | 4373117 | 28.86 | 0.80 | 0.0869 |  |
| hsa-miR-181b | 4373116 | 30.69 | 0.77 | 0.1399 |  |
| **hsa-miR-181c** | **4373115** | **31.69** | **0.35** | **0.0177** | ***** |
| hsa-miR-181d | 4373180 | 32.56 | 1.28 | 0.6109 |  |
| hsa-miR-182 | 4373271 | 31.43 | 1.02 | 0.6846 |  |
| hsa-miR-183 | 4373114 | 34.01 | 2.36 | 0.0827 |  |
| hsa-miR-184 | 4373113 | 26.23 | 0.80 | 0.2233 |  |
| hsa-miR-186 | 4373112 | 30.53 | 1.05 | 0.9061 |  |
| **hsa-miR-187** | **4373111** | **30.82** | **0.77** | **0.0181** | ***** |
| hsa-miR-188 | 4373182 | 32.40 | 1.19 | 0.4199 |  |
| hsa-miR-18a | 4373118 | 34.04 | 2.76 | 0.9393 |  |
| hsa-miR-190 | 4373110 | 34.44 | 0.58 | 0.2449 |  |
| hsa-miR-191 | 4373109 | 26.16 | 1.00 | 0.9868 |  |
| hsa-miR-192 | 4373108 | 32.77 | 1.06 | 0.9815 |  |
| hsa-miR-194 | 4373106 | 32.46 | 0.78 | 0.1552 |  |
| hsa-miR-195 | 4373105 | 30.22 | 0.78 | 0.0713 |  |
| hsa-miR-19a | 4373099 | 30.95 | 0.79 | 0.1071 |  |
| hsa-miR-19b | 4373098 | 27.78 | 0.95 | 0.6945 |  |
| hsa-miR-200a | 4378069 | 27.03 | 0.79 | 0.3599 |  |
| hsa-miR-203 | 4373095 | 34.51 | 0.69 | 0.3460 |  |
| hsa-miR-204 | 4373094 | 29.92 | 0.88 | 0.2727 |  |
| hsa-miR-20a | 4373286 | 30.94 | 1.04 | 0.9541 |  |
| **hsa-miR-21** | **4373090** | **32.57** | **0.30** | **0.0192** | ***** |
| hsa-miR-210 | 4373089 | 28.04 | 0.85 | 0.4148 |  |
| hsa-miR-212 | 4373087 | 28.14 | 0.98 | 0.8320 |  |
| hsa-miR-213 | 4373086 | 30.88 | 1.38 | 0.7182 |  |
| hsa-miR-218 | 4373081 | 25.71 | 0.84 | 0.0869 |  |
| hsa-miR-22 | 4373079 | 29.61 | 0.86 | 0.5468 |  |
| hsa-miR-221 | 4373077 | 30.91 | 1.22 | 0.9483 |  |
| hsa-miR-222 | 4373076 | 29.92 | 0.97 | 0.7400 |  |
| hsa-miR-23b | 4373073 | 31.62 | 0.82 | 0.5960 |  |
| hsa-miR-24 | 4373072 | 25.91 | 0.92 | 0.5405 |  |
| hsa-miR-26a | 4373070 | 26.80 | 0.95 | 0.6482 |  |
| hsa-miR-26b | 4373069 | 30.08 | 1.14 | 0.6170 |  |
| hsa-miR-27a | 4373287 | 32.88 | 0.66 | 0.4095 |  |
| hsa-miR-27b | 4373068 | 32.57 | 0.80 | 0.2870 |  |
| hsa-miR-296 | 4373066 | 28.90 | 0.95 | 0.7068 |  |
| hsa-miR-299-5p | 4373188 | 34.32 | 0.76 | 0.4221 |  |
| hsa-miR-29a | 4373065 | 28.06 | 0.74 | 0.1528 |  |
| hsa-miR-29b | 4373288 | 32.31 | 2.25 | 0.6688 |  |
| hsa-miR-29c | 4373289 | 30.95 | 0.92 | 0.5853 |  |
| **hsa-miR-301** | **4373064** | **29.92** | **0.74** | **0.0413** | ***** |
| hsa-miR-302a | 4378070 | 30.76 | 1.82 | 0.4714 |  |
| hsa-miR-30a-3p | 4373062 | 29.70 | 0.87 | 0.3321 |  |
| hsa-miR-30a-5p | 4373061 | 28.55 | 0.87 | 0.3385 |  |
| **hsa-miR-30b** | **4373290** | **26.65** | **0.83** | **0.0038** | ****** |
| **hsa-miR-30c** | **4373060** | **25.59** | **0.74** | **0.0430** | ***** |
| hsa-miR-30d | 4373059 | 29.55 | 0.90 | 0.3455 |  |
| hsa-miR-30e-5p | 4373058 | 32.53 | 0.96 | 0.8555 |  |
| hsa-miR-32 | 4373056 | 35.29 | 0.80 | 0.3822 |  |
| hsa-miR-320 | 4373055 | 30.99 | 0.71 | 0.2563 |  |
| hsa-miR-324-3p | 4373053 | 29.78 | 1.08 | 0.7168 |  |
| hsa-miR-324-5p | 4373052 | 31.73 | 0.78 | 0.3636 |  |
| hsa-miR-328 | 4373049 | 25.75 | 0.86 | 0.1870 |  |
| hsa-miR-331 | 4373046 | 27.91 | 0.97 | 0.7735 |  |
| hsa-miR-335 | 4373045 | 30.86 | 0.84 | 0.1615 |  |
| hsa-miR-339 | 4373042 | 34.82 | 2.28 | 0.3154 |  |
| hsa-miR-340 | 4373041 | 30.97 | 0.59 | 0.0549 |  |
| hsa-miR-342 | 4373040 | 27.88 | 1.08 | 0.6885 |  |
| hsa-miR-34a | 4373278 | 34.93 | 1.16 | 0.6920 |  |
| hsa-miR-34c | 4373036 | 31.74 | 0.25 | 0.1145 |  |
| **hsa-miR-361** | **4373035** | **30.51** | **0.27** | **0.0149** | ***** |
| **hsa-miR-365** | **4373194** | **31.85** | **0.71** | **0.0330** | ***** |
| hsa-miR-375 | 4373027 | 32.64 | 1.05 | 0.7850 |  |
| hsa-miR-376a# | 4378104 | 33.05 | 0.74 | 0.1755 |  |
| hsa-miR-378 | 4373024 | 33.89 | 0.59 | 0.3343 |  |
| hsa-miR-379 | 4373023 | 30.79 | 0.97 | 0.6677 |  |
| hsa-miR-380-5p | 4373021 | 34.09 | 1.21 | 0.8056 |  |
| hsa-miR-382 | 4373019 | 28.77 | 0.72 | 0.0693 |  |
| hsa-miR-412 | 4373199 | 30.12 | 0.80 | 0.5533 |  |
| hsa-miR-422b | 4373016 | 31.44 | 1.49 | 0.3504 |  |
| hsa-miR-423 | 4373015 | 34.45 | 1.99 | 0.4401 |  |
| **hsa-miR-433** | **4373205** | **28.95** | **0.79** | **0.0405** | ***** |
| hsa-miR-449 | 4373207 | 34.48 | 0.57 | 0.2981 |  |
| hsa-miR-455 | 4378098 | 31.32 | 1.25 | 0.9374 |  |
| hsa-miR-484 | 4381032 | 26.94 | 1.06 | 0.8579 |  |
| hsa-miR-485-5p | 4373212 | 29.59 | 0.90 | 0.6023 |  |
| hsa-miR-486 | 4378096 | 29.64 | 1.12 | 0.6678 |  |
| hsa-miR-493-3p | 4378100 | 31.72 | 1.10 | 0.9296 |  |
| hsa-miR-532 | 4380928 | 29.80 | 0.81 | 0.3308 |  |
| hsa-miR-539 | 4378103 | 29.57 | 0.90 | 0.1181 |  |
| hsa-miR-542-3p | 4378101 | 32.20 | 1.53 | 0.2638 |  |
| hsa-miR-7 | 4373014 | 31.06 | 0.96 | 0.7495 |  |
| **hsa-miR-9** | **4373285** | **24.17** | **0.76** | **0.0115** | ***** |
| hsa-miR-9# | 4378074 | 25.42 | 0.85 | 0.1679 |  |
| hsa-miR-92 | 4373013 | 34.60 | 1.54 | 0.1586 |  |
| hsa-miR-98 | 4373009 | 33.18 | 0.67 | 0.3024 |  |
| hsa-miR-99a | 4373008 | 27.73 | 0.73 | 0.1976 |  |
| hsa-miR-99b | 4373007 | 29.73 | 0.84 | 0.1873 |  |
| mmu-let-7d# | 4378108 | 32.56 | 0.20 | 0.0643 |  |
| mmu-miR-101b | 4373292 | 30.29 | 0.97 | 0.7871 |  |
| mmu-miR-106a | 4373293 | 33.74 | 0.65 | 0.2020 |  |
| mmu-miR-124a | 4373295 | 27.71 | 0.79 | 0.0644 |  |
| mmu-miR-127 | 4373296 | 26.88 | 0.95 | 0.6809 |  |
| mmu-miR-129-3p | 4373297 | 25.85 | 0.84 | 0.2136 |  |
| mmu-miR-129-5p | 4373298 | 31.78 | 1.25 | 0.6569 |  |
| mmu-miR-134 | 4373299 | 31.49 | 1.04 | 0.9670 |  |
| **mmu-miR-137** | **4373301** | **27.75** | **0.79** | **0.0127** | ***** |
| mmu-miR-140 | 4373374 | 29.05 | 0.88 | 0.2645 |  |
| mmu-miR-151 | 4373304 | 30.56 | 0.69 | 0.0771 |  |
| mmu-miR-153 | 4373305 | 33.25 | 4.09 | 0.4466 |  |
| mmu-miR-155 | 4381094 | 33.93 | 1.56 | 0.7066 |  |
| mmu-miR-187 | 4373307 | 28.97 | 0.82 | 0.3941 |  |
| mmu-miR-192 | 4373308 | 33.11 | 1.74 | 0.9565 |  |
| mmu-miR-202 | 4373311 | 33.71 | 2.97 | 0.4676 |  |
| mmu-miR-204 | 4373313 | 29.99 | 0.90 | 0.5320 |  |
| **mmu-miR-20b** | **4378121** | **32.71** | **0.28** | **0.0188** | ***** |
| mmu-miR-211 | 4373315 | 31.47 | 1.31 | 0.8795 |  |
| mmu-miR-221 | 4373318 | 28.98 | 0.93 | 0.5005 |  |
| mmu-miR-291-5p | 4373322 | 29.84 | 0.77 | 0.3572 |  |
| mmu-miR-300 | 4373330 | 33.09 | 1.31 | 0.5827 |  |
| mmu-miR-31 | 4373331 | 29.46 | 0.95 | 0.6522 |  |
| mmu-miR-324-5p | 4373333 | 29.66 | 0.86 | 0.0915 |  |
| mmu-miR-326 | 4373335 | 32.43 | 0.99 | 0.8512 |  |
| mmu-miR-329 | 4373336 | 32.72 | 0.77 | 0.4285 |  |
| mmu-miR-330 | 4373337 | 28.80 | 1.18 | 0.4061 |  |
| mmu-miR-337 | 4373338 | 30.69 | 0.57 | 0.0546 |  |
| mmu-miR-344 | 4373340 | 31.70 | 0.81 | 0.2182 |  |
| mmu-miR-345 | 4373341 | 33.73 | 1.02 | 0.7205 |  |
| mmu-miR-350 | 4373344 | 32.53 | 1.20 | 0.4514 |  |
| mmu-miR-362 | 4386755 | 34.03 | 0.66 | 0.4217 |  |
| mmu-miR-369-5p | 4378118 | 29.81 | 0.79 | 0.1071 |  |
| mmu-miR-374-5p | 4381045 | 30.05 | 1.04 | 0.7904 |  |
| mmu-miR-376a | 4373347 | 29.09 | 0.90 | 0.4380 |  |
| **mmu-miR-376b** | **4373348** | **32.68** | **2.61** | **0.0256** | ***** |
| mmu-miR-376c | 4378112 | 32.77 | 1.40 | 0.2621 |  |
| mmu-miR-379 | 4373349 | 29.37 | 0.76 | 0.2205 |  |
| mmu-miR-383 | 4381093 | 33.09 | 0.70 | 0.1472 |  |
| mmu-miR-384 | 4373352 | 34.16 | 2.00 | 0.7905 |  |
| **mmu-miR-409** | **4373353** | **33.37** | **0.26** | **0.0200** | ***** |
| mmu-miR-434-3p | 4373358 | 25.81 | 0.81 | 0.1270 |  |
| mmu-miR-434-5p | 4373359 | 32.25 | 0.95 | 0.7099 |  |
| mmu-miR-449b | 4386747 | 32.89 | 2.32 | 0.9013 |  |
| mmu-miR-451 | 4373360 | 25.82 | 0.76 | 0.2635 |  |
| mmu-miR-467# | 4381084 | 32.61 | 1.27 | 0.3004 |  |
| mmu-miR-467b | 4381092 | 32.22 | 1.62 | 0.4526 |  |
| mmu-miR-485-3p | 4386764 | 29.83 | 1.01 | 0.9946 |  |
| mmu-miR-487b | 4378116 | 27.77 | 0.84 | 0.1031 |  |
| mmu-miR-488# | 4381074 | 33.80 | 0.92 | 0.6584 |  |
| mmu-miR-491 | 4381053 | 29.74 | 0.93 | 0.3813 |  |
| mmu-miR-495 | 4381078 | 29.66 | 0.91 | 0.2187 |  |
| mmu-miR-496 | 4386771 | 33.53 | 2.97 | 0.3784 |  |
| mmu-miR-497 | 4381046 | 31.22 | 0.96 | 0.7490 |  |
| mmu-miR-501# | 4381069 | 29.93 | 0.88 | 0.5867 |  |
| mmu-miR-540 | 4378119 | 32.40 | 2.99 | 0.2067 |  |
| mmu-miR-541 | 4378113 | 31.13 | 0.81 | 0.4388 |  |
| mmu-miR-543 | 4378111 | 30.72 | 0.72 | 0.1459 |  |
| mmu-miR-666 | 4386770 | 31.05 | 1.31 | 0.2173 |  |
| mmu-miR-667 | 4386769 | 29.32 | 0.91 | 0.5265 |  |
| mmu-miR-668 | 4386767 | 30.80 | 0.95 | 0.7138 |  |
| mmu-miR-669a | 4381091 | 31.14 | 0.58 | 0.2333 |  |
| mmu-miR-673 | 4386772 | 32.90 | 0.88 | 0.7265 |  |
| mmu-miR-674# | 4386773 | 31.67 | 1.35 | 0.1783 |  |
| mmu-miR-676 | 4386776 | 31.17 | 1.12 | 0.9046 |  |
| mmu-miR-678 | 4381076 | 31.93 | 0.57 | 0.1317 |  |
| mmu-miR-680 | 4381079 | 30.42 | 0.84 | 0.3508 |  |
| mmu-miR-685 | 4386748 | 28.91 | 1.42 | 0.0803 |  |
| mmu-miR-687 | 4386750 | 26.62 | 1.25 | 0.4086 |  |
| mmu-miR-690 | 4381086 | 30.52 | 0.77 | 0.1897 |  |
| mmu-miR-692 | 4381088 | 30.83 | 0.84 | 0.4592 |  |
| mmu-miR-694 | 4381090 | 34.45 | 2.89 | 0.0637 |  |
| mmu-miR-696 | 4381051 | 27.30 | 1.52 | 0.4220 |  |
| mmu-miR-698 | 4381055 | 31.92 | 0.96 | 0.8748 |  |
| mmu-miR-699 | 4381056 | 29.09 | 0.77 | 0.4074 |  |
| **mmu-miR-700** | **4381057** | **32.10** | **0.55** | **0.0470** | ***** |
| mmu-miR-702 | 4381059 | 31.35 | 0.95 | 0.7781 |  |
| mmu-miR-704 | 4386745 | 31.41 | 0.63 | 0.1024 |  |
| mmu-miR-706 | 4381061 | 29.72 | 0.83 | 0.3451 |  |
| mmu-miR-709 | 4381063 | 23.52 | 1.08 | 0.5860 |  |
| mmu-miR-715 | 4381067 | 30.95 | 0.55 | 0.3136 |  |
| mmu-miR-720 | 4381052 | 28.32 | 0.93 | 0.5898 |  |
| mmu-miR-721 | 4381073 | 35.10 | 1.13 | 0.8434 |  |
| mmu-miR-7b | 4373370 | 31.47 | 1.27 | 0.8563 |  |
| mmu-miR-9 | 4373371 | 25.65 | 0.80 | 0.0646 |  |
| mmu-miR-93 | 4373302 | 30.53 | 0.74 | 0.1097 |  |
| mmu-miR-99a | 4373373 | 27.15 | 0.88 | 0.3489 |  |
| rno-miR-1 | 4381125 | 30.76 | 1.05 | 0.9410 |  |
| rno-miR-207 | 4381096 | 29.54 | 0.93 | 0.6027 |  |
| rno-miR-20b | 4381106 | 32.48 | 0.54 | 0.2078 |  |
| rno-miR-29c# | 4381131 | 33.19 | 1.48 | 0.1470 |  |
| rno-miR-327 | 4381108 | 32.50 | 0.32 | 0.0560 |  |
| rno-miR-346 | 4381113 | 30.32 | 0.56 | 0.1189 |  |
| rno-miR-350 | 4381117 | 31.65 | 1.55 | 0.6503 |  |
| rno-miR-352 | 4381119 | 32.59 | 0.99 | 0.7047 |  |
| rno-miR-381 | 4381102 | 32.17 | 1.33 | 0.9625 |  |
| rno-miR-382# | 4381128 | 30.47 | 0.55 | 0.2634 |  |
| rno-miR-422b | 4381095 | 30.52 | 0.73 | 0.1583 |  |
| **rno-miR-664** | **4381103** | **29.79** | **0.76** | **0.0379** | ***** |
| rno-miR-7 | 4378122 | 32.57 | 0.73 | 0.3091 |  |
| rno-miR-7# | 4381118 | 30.11 | 0.85 | 0.4247 |  |
| snoRNA135 | 4380912 | 28.80 | 0.88 | 0.1911 |  |
| U87 | 4386735 | 31.12 | 0.64 | 0.0809 |  |

Expression profiling of microRNAs in mouse primary hippocampal cells with and without Aβ42 treatment using Rodent TaqMan Low Denisty miRNA Arrays. Shown are the results for 230 miRNAs in ascending order out of the 381 miRNAs present on the TLDA whose amplification plots where above the cutoff threshold in the triplicate analysis. miRNA expression levels can be gauged using Average (Ave) C*t* values. miRNAs highlighted in bold are those significantly deregulated. T-test P-value significance: **P<0.01, *P<0.05.
